# Concurrent sexual partnerships do not explain the HIV epidemics in Africa: a systematic review of the evidence

**DOI:** 10.1186/1758-2652-13-34

**Published:** 2010-09-13

**Authors:** Larry Sawers, Eileen Stillwaggon

**Affiliations:** 1 Department of Economics, American University, Washington, DC USA; 2 Department of Economics, Gettysburg College, Gettysburg, PA USA

## Abstract

The notion that concurrent sexual partnerships are especially common in sub-Saharan Africa and explain the region's high HIV prevalence is accepted by many as conventional wisdom. In this paper, we evaluate the quantitative and qualitative evidence offered by the principal proponents of the concurrency hypothesis and analyze the mathematical model they use to establish the plausibility of the hypothesis.

We find that research seeking to establish a statistical correlation between concurrency and HIV prevalence either finds no correlation or has important limitations. Furthermore, in order to simulate rapid spread of HIV, mathematical models require unrealistic assumptions about frequency of sexual contact, gender symmetry, levels of concurrency, and per-act transmission rates. Moreover, quantitative evidence cited by proponents of the concurrency hypothesis is unconvincing since they exclude Demographic and Health Surveys and other data showing that concurrency in Africa is low, make broad statements about non-African concurrency based on very few surveys, report data incorrectly, report data from studies that have no information about concurrency as though they supported the hypothesis, report incomparable data and cite unpublished or unavailable studies. Qualitative evidence offered by proponents of the hypothesis is irrelevant since, among other reasons, there is no comparison of Africa with other regions.

Promoters of the concurrency hypothesis have failed to establish that concurrency is unusually prevalent in Africa or that the kinds of concurrent partnerships found in Africa produce more rapid spread of HIV than other forms of sexual behaviour. Policy makers should turn attention to drivers of African HIV epidemics that are policy sensitive and for which there is substantial epidemiological evidence.

## Introduction

Prevalence of HIV in some countries of sub-Saharan Africa is up to 50 times higher than the average for countries outside Africa. In the 1990s, it was widely accepted in policy and scholarly discourse that higher rates of risky sexual behaviour in Africa explained the difference in HIV prevalence. That conventional wisdom was repeated in hundreds of articles, books and policy documents, as well as in popular media. Careful examination of empirical evidence, however, compelled social scientists and policy makers alike to acknowledge that most kinds of risky sexual behaviours are not exceptionally common in sub-Saharan Africa [1-5]. On the contrary, rates of risky behaviours are considerably higher in affluent and middle-income countries with low HIV prevalence, including early initiation of sex, number of sexual partners, and premarital and extramarital sexual relations [5-18]. Confronted with that evidence, defenders of the notion that some form of risky sexual behaviour must explain the high HIV prevalence in sub-Saharan Africa narrowed their argument to a single kind of sexual behaviour: concurrency, which they define as long-term, overlapping partnerships.

The concurrency hypothesis consists of two claims: that concurrency leads to more rapid spread of HIV than other forms of heterosexual partnering and that concurrency is more prevalent in eastern and southern Africa than in the rest of the world. The concurrency hypothesis is about the difference between Africa and the rest of the world. The focus of the concurrency hypothesis and this article is not the distribution of HIV risk within countries, but rather the factor or factors that have shifted the entire distribution of risk for populations in eastern and southern Africa. The proponents of the concurrency hypothesis have correctly conceded that other forms of multiple partnering are less common in sub-Saharan Africa than elsewhere [19,20]. Whether concurrency is much higher in Africa than elsewhere and, consequently, whether it is the appropriate focus of HIV-prevention policy remain in contention.

This paper focuses on the work of the four principal proponents of the concurrency hypothesis: Daniel Halperin, Helen Epstein, Martina Morris and Timothy Mah. The most complete defence of the hypothesis is a six-page article by Mah and Halperin [21]. In addition, Halperin and Epstein have published two short papers, and Epstein devoted several pages of her book and a short article to an explanation of the concurrency hypothesis [19,20,22,23]. They have relied heavily on the mathematical modelling of Martina Morris and Mirjam Kretzschmar in their attempt to show the plausibility of the hypothesis [24-26]. These articles promoting the concurrency hypothesis have been cited in scholarly works more than 800 times, according to Google Scholar. In the past five years, the hypothesis that "concurrency is a key driver of the epidemics in southern and parts of east Africa" [27] has become the new conventional wisdom.

The concurrency hypothesis has its critics [28,29], most notably Mark Lurie and Samantha Rosenthal [30-32], to which Mah, Halperin, Morris and Epstein [27,33,34] have responded. Kretzschmar [35] has recently argued that some forms of concurrent partnerships may be protective. The purpose of the present article is to build on the work of those critics, producing a more thorough examination of the model and the empirical evidence offered in support of the concurrency hypothesis.

The only direct way to establish the importance of concurrency in spreading HIV would be to map sexual networks by interviewing all partners to determine if people whose partners have other partners are more likely to become infected with HIV than people whose partners have no other partner. Helleringer *et al *tried to test the concurrency hypothesis in that way, but in only 23% of concurrent partnerships were both partners tested for HIV [36,37]. The resulting sample selection bias severely limits the ability to make useful statistical inferences from their data. Moreover, that study did not distinguish between long-term overlapping partnerships and other forms of multiple partnering. The researchers also measured prevalence (net accumulated infections), not incidence (new infections), and did not genetically sequence the virus in infected partners to verify the source of their infection. Finally, the study was conducted on remote Likoma Island. The nearly closed nature of the island network allowed it to be mapped thoroughly, but that very advantage, as Lurie and Rosenthal point out [31], limits its applicability to other situations. Moreover, the study of sexual networks within a community does not necessarily tell us anything about the concurrency hypothesis, which compares a group of countries to the rest of the world.

Ecological studies have looked for population correlations between HIV prevalence and the rate of self-reported concurrency. Mah, Halperin and Morris reject ecological studies because they do not measure the partner's concurrency status and they measure prevalence of HIV, not incidence [27,34]. Nevertheless, the concurrency hypothesis is itself an ecological assertion (about two characteristics of populations). Ultimately, the lack of correlation between HIV and concurrency seriously undermines the hypothesis. Morris argues that "the correlation ... will only be present when both HIV prevalence and behavior have been at equilibrium for some time. That is not the case in any sub-Saharan African country" [34]. The stability of both HIV prevalence and sexual behaviour in most countries in Africa, however, suggests that the situation is close enough to equilibrium to expect a correlation, if concurrency were in fact a driver of the epidemics. As Lurie and Rosenthal point out, there have been at least four ecological studies of HIV and concurrency, and none finds a statistically significant correlation between rates of self-reported concurrency and HIV prevalence [38-41]. In addition, three other studies show that individuals in concurrent partnerships are no more likely to be infected with HIV than those who are not [42-44].

Given that all of the studies that have looked for a statistical correlation between concurrency and HIV either have important limitations (Likoma) or find no correlation (all the rest), the proponents of the concurrency hypothesis have had to turn to other evidence to make their case [19-23]. First, they argue that mathematical modelling shows that HIV can spread far more rapidly if long-term, overlapping partnerships are common compared with situations where multiple partnering is confined to serial monogamy. Second, they offer quantitative evidence that they claim shows a higher level of concurrency in sub-Saharan Africa than elsewhere. Last, they present qualitative evidence about attitudes, perceptions and beliefs to support their argument. We examine each of those categories in turn. Mah and Halperin argue that the "totality of the evidence" is persuasive [27]. Evaluating each argument and each datum, we find that the "totality of the evidence" does not support the hypothesis.

## The model

Mathematical models of disease transmission are key to establishing the validity of the concurrency hypothesis. Even if its proponents could show that concurrency was more common in sub-Saharan Africa than elsewhere, the hypothesis would not be viable unless it were plausible that concurrency could produce a substantially more rapid spread of HIV than non-concurrency.

Halperin, Epstein and Mah [19-23] rely on the stochastic simulation model of HIV transmission produced by Morris and Kretzschmar [24-26]. Their model permits two kinds of partnerships: concurrent and serially monogamous. For the duration of a serially monogamous partnership, neither partner has sex outside the partnership. Concurrent partnerships are those in which partners have other long-term partnerships. The length of partnerships is determined randomly and averages six to seven months in the model's early versions and about two to 20 years in their latest version. The model is seeded with 20 randomly distributed HIV infections and is then simulated for 1825 iterations (the number of days in five years). They report the average number of HIV cases in 100 simulations. Next, they repeat the process, varying the proportion of partnerships that are concurrent, holding the total number of partnerships constant. The goal is to see how different levels of concurrency affect the number of new infections in the population generated by the simulations.

Any model is only as good as its algorithm and its assumptions about the model's parameters. Their model makes assumptions about: (1) the frequency of sexual contact in partnerships; (2) the concurrency rates of men compared with women; (3) the per-act transmission rate of HIV; and (4) the level of concurrency. We will examine each of those assumptions in turn and show that the parameters are without empirical support.

### Frequency of sexual contact

As Lurie and Rosenthal [31,32] point out, Morris and Kretzschmar [24-26] assume that everyone in a partnership has sexual contact with every one of their partners every day (page 182 in reference [24] and page 645 in reference [25]). If they had modelled weekly instead of daily sex, it would take 35 years instead of five years to reach the same differential in HIV cases between concurrency and only serial monogamy. (They say, "the simulation time step is 1 day" [24], but calling the time period between iterations a "day" is purely arbitrary. One could just as easily call the time step an hour, a week, or a month. If we call it a week, then 1825 weekly iterations of the model take 35 years, which would also lengthen the duration of partnerships commensurately.) If people in their model had sex every month, it would take 152 years for the trajectories of the epidemics, with and without concurrency, to diverge by the same amount as produced by five years of daily sex.

Most people do not have sex every day with multiple partners, and Morris and Kretzschmar do not and cannot justify their assumption of daily sex. Halperin and Mah do not address the issue, even in response to Lurie and Rosenthal's criticism [27,31]. Epstein argues that reducing the frequency of sex "would have slowed down the epidemic, but would not substantially affect the comparison between serial monogamy and concurrency" [33]. Slowing down the epidemic from five years to 152 (or even 35) years, of course, eliminates any practical support that Morris and Kretzschmar's model can provide for the concurrency hypothesis. We present here empirical evidence from 13 studies showing far lower frequency of sexual contact in Africa than Morris and Kretzschmar assume, even in age groups usually reported to be most sexually active. Note that Halperin, Epstein and Mah cite five of the following studies, but do not report the data in those studies that show how infrequently many people in sub-Saharan Africa have sex with their ongoing partners.

• Lurie and Rosenthal [31] cite a study of South African sexually experienced men and women aged 15-24 years [45] in which 90% or more reported having had sex fewer than five times in the previous month.

• Caraël [46] reports that in Lesotho, Tanzania, Togo, Burundi and Côte d'Ivoire, between 32% and 59% of adults with regular partners reported no sex with their regular partner in the previous month (page 104 in reference [46]). In those countries, mean coital frequency with regular partners in the previous month was 4.0 for adult men and 3.2 for adult women.

• Jewkes *et al *[42] report on a study of rural South African sexually experienced men aged 15-26 in Eastern Cape Province, 89% of whom had a regular girlfriend (page 1458 in reference [42]). Half of the young men had not had sex in the previous three weeks and one-quarter had not had sex in the previous 60 days.

• Harrison *et al *[47] report that among sexually experienced youth aged 15-24 in rural KwaZulu-Natal in South Africa, 53.9% of men and 81.8% of women reported no sex with their most recent partner in the previous week (page 301 in reference [47]). The figures for no sex in the previous month were 27.2% for men and 56.4% for women.

• Cleland and Ali [48], using data from 18 African countries, report that the median percentage of sexually experienced unmarried women aged 15-24 who had no sex in the previous three months was 49.2.

• A nationwide study in South Africa [49] reports that among youth aged 15-24 who were sexually experienced, 23% of men and 20% of women had not had sex in the previous year (Table 3.22 in reference [49]). Among those who did have sex in the previous year, 24.3% reported no sex in the previous month and more than 70% reported having sex fewer than five times in the previous month (Figure 3.15 in reference [49]).

• Brewis and Meyer [50] report data from the Demographic and Health Surveys (DHS) for nine sub-Saharan African countries (Table Four in reference [50]). They find that the frequency of sex among women in their first year of marriage ranged from two times a month in Mali and Burkina Faso to 4.4 times per month in Malawi and averaged 3.2 times per month. Coital frequency in later years of marriage was much lower.

• The 2002 Reproductive Health Survey in Lesotho [51] reports that 57.4% of sexually experienced adults (men aged 12 to 54 and women aged 12 to 49) did not have sex in the previous four weeks (Table 3.11 in reference [51]). Among married respondents, 46.4% had not had sex in the previous four weeks and the mean coital frequency in those four weeks was 6.1 (Table 3.16 in reference [51]).

• A study of Kenyan truckers [52], 77% of whom were married, reported that half had sex fewer than four times per month (page 548 in reference [52]).

• Gourvenec *et al *[53] reported that 20% of sexually experienced women aged 15-34 in Botswana were "still having sex with" two or more ongoing partners at the time of the interview (Table Four in reference [53]). Only 6% of sexually experienced women had sex with more than one partner in the previous month; therefore, almost all of the women did not have sex with their second or third ongoing partner in the previous month (Table Five in reference [53]).

• Stewart *et al *[54], using data from a DHS survey in 1994-1995, report that only 43% of married women in the Central African Republic had sex more often than four times in the previous month (Figure Four in reference [54]). Only one woman out of 2188 reported sex as often as twice a day (page 531 in reference [54]).

• Using data from four different nationally representative DHS surveys between 1991 and 1999, a study of adult Tanzanians [55] shows that, on average, 33% of men and 40% of women did not have sex in the previous month and 17% of men and 25% of women did not have sex in the previous year (Table C3 in reference [55]).

• In a South African study of more than 3000 sexually active men aged 18 to 24 [56], respondents reported sexual contact every 46 to 55 days on average in the nine months prior to the interview (page 2 in reference [56]). (The interviews were conducted a year after the men had been randomly distributed between two groups: those who were circumcised immediately and those who were to be circumcised after the interview.)

Further evidence that Morris and Kretzschmar overestimate the frequency of sexual contact is common sense. In Africa, as everywhere, people can be in a regular sexual relationship (going steady) and not have sex. That is particularly true for young people [5]. Even sexually active people have jobs, kids to raise, and laundry and homework to do. They might sleep in the same room as their parents, children or siblings. They are tired at the end of the day. Moreover, advocates of the concurrency hypothesis say that long-distance labour migration predisposes sub-Saharan Africans to concurrency [21]. Neither the miner in South Africa nor the office worker in Lilongwe goes home to his wife in the Malawian countryside every day. In short, the assumption of sex every day with every partner, which is critical to how rapidly the model generates new HIV infections, does not reflect reality.

### Gender symmetry

A second parameter that drives early versions of Morris and Kretzschmar's model and produces exponential growth in HIV is the assumption of gender symmetry: that is, men and women's rates of concurrency are equal [24,25]. Halperin and Epstein argue that "as soon as one person in a network of concurrent relationships contracts HIV, everyone else in the network is placed at risk. By contrast, serial monogamy traps the virus within a single relationship for months or years" [19]. Nevertheless, if women do not have concurrent partners, then HIV infection is "trapped" in the same way that it is with serial monogamy, blocking the formation of "extensive interlocking sexual networks" [19]. Halperin and Epstein acknowledge the importance of gender symmetry when they state that large-scale heterosexual networks can only emerge when a "significant proportion of women are engaging in multiple longer-term partnerships" [20]. As will become clearer in the following discussion of data on concurrency, every survey in Africa that has measured women's concurrency has found that women are far less likely to have concurrent partners than men.

In their 2000 article, Morris and Kretzschmar abandon the assumption of gender symmetry [26]. They calibrate the model still assuming sex every day with every partner, but use rates of concurrency of men and women (and length of partnerships) from the 1994 Ugandan Sexual Network Study of the Rakai district of Uganda. They find that, after 1825 iterations of their model, the number of HIV cases is on average only 26% higher than with no concurrency (page 124 in reference [26]). Their earlier model, which assumes gender symmetry (Figure Three, page 645, in reference [25]), generates about 100% more cases of HIV assuming the same level of concurrency as in Rakai [25]. (See Appendix A for an explanation.) In other words, eliminating the assumption of gender symmetry (and also lengthening the duration of the average partnership) reduces the impact of concurrency on HIV prevalence by about 75% when concurrency is at the same level as in Rakai.

### Transmission rates

Morris and Kretzschmar [24-26] assume a per-act transmission rate of 0.05. To illustrate the importance of the per-act transmission rate for the modelling of HIV, Deuchert and Brody show that with the level of concurrency found in Rakai and the generally accepted transmission rate of 0.001, every adult in Rakai would have to have 47 sex acts per day per partner to reproduce Morris and Kretzschmar's results [28]. Lurie and Rosenthal extend that criticism by arguing that after the first few weeks of acute infection, the transmission rate drops to levels far below the rate used by Morris and Kretzschmar. Lurie and Rosenthal add without explanation that lower transmission rates would reduce the contrast in HIV infections between concurrency and serial monogamy [31]. Epstein disagrees, saying, "While the transmission rates used by Morris and Kretzschmar are high, this does not affect the comparison between concurrency and serial monogamy, since both would be affected by changes in the transmission rate" [33]. Epstein's assertion is incorrect, as we demonstrate mathematically in Appendix B. Lowering the transmission rate must reduce the contrast between concurrency and serial monogamy.

Numerous studies have found that between heterosexuals who are otherwise healthy, the per-act transmission rate during asymptomatic infection (after the brief acute infection period) is about one in 1000 contacts [28,57-61]. Even that transmission rate may be an overestimate since some studies did not control for such factors as blood exposures, cofactor infections or anal intercourse [61]. During acute infection, no one has found a heterosexual transmission rate as high as 0.05 in the absence of cofactor infection [59,62-64]. Morris and Kretzschmar (page 116 in reference [26]) took their 0.05 transmission rate from a study of Thai soldiers by Mastro *et al *[65]. That study also reports the widely accepted 0.001 transmission rate between otherwise healthy adults. Morris and Kretzschmar do not point out that 43% of the Thai soldiers were afflicted with other sexually transmitted infections (STIs) that are known to elevate transmission rates, and that the objective of the Thai study was to explain the anomalously high transmission rate between the soldiers and commercial sex workers, 50 times higher than between otherwise healthy adults.

Epstein's argument is exactly backwards when she says, "Morris and Kretzschmar's models do not include [the early peak in infectivity], so one could argue they actually underestimate the relative effect of concurrency" [33]. Morris and Kretzschmar do, in fact, include high early infectivity in their model. The 0.05 transmission rate used by Morris and Kretzschmar is even higher than any research has found during acute infection in the absence of cofactor infections. What Morris and Kretzschmar did not model was *lower *(indeed, dramatically lower) transmission rates *after *the period of acute infection. That failure to model realistically low transmission rates during acute infection and vanishingly small transmission rates during asymptomatic infection overstates the effect of concurrency.

In all three versions of their model, Morris and Kretzschmar assume a 0.05 transmission rate and daily sex [24-26]. With the daily sex assumption in place, it does not make any important difference to the outcome whether or not one follows Lurie and Rosenthal's suggestion to use a lower transmission rate after acute infection. That is because, given the model's assumptions, most people will become infected during a partner's period of acute infection, even with realistic transmission rates that are far lower than 0.05. (See Appendix C for an explanation.) If, however, one assumes a frequency of sexual contact consistent with the evidence presented here, then few new infections will occur during acute infection, after which the transmission rate decreases to levels that cannot sustain the epidemic spread of HIV [64,66]. Thus, the combined assumptions of frequency of sex and high transmission rate make it appear as if concurrency can produce a much more rapid spread of HIV infections than other sexual behaviour.

### Levels of concurrency

In both of their joint articles, Halperin and Epstein [19,20] say that Morris and Kretzschmar's model shows that "with long-term concurrency ... the resulting epidemic was ten times greater" than without any concurrent partnerships [19]. They cite the 1997 version of Morris and Kretzschmar's model, which produces a 10-fold difference in the epidemics only by assuming that half of partnerships are concurrent [25]. At levels of concurrency close to what actually prevail in most of sub-Saharan Africa, however, the model shows that the number of HIV infections is between 30% and 100% larger (assuming daily sex, a 0.05 transmission rate, and gender symmetry) than with only serial monogamy (Figure Three, page 645, in reference [25]), not 1000% larger as reported by Halperin and Epstein.

Mah and Halperin (page 12 in reference [21]) repeat the 10-times-as-much claim, citing the 2000 version of Morris and Kretzschmar's model [26]. They incorrectly state that the 10-fold figure is based on "data from Uganda". As noted, the model using data from Rakai, Uganda (where 20.2% of partnerships were concurrent and with gender asymmetry), produces only 26% more HIV cases after 1825 iterations compared to only serial monogamy [26]. That extra 26% does little to explain sub-Saharan Africa's extraordinary HIV prevalence. Outside the region, HIV prevalence averages 0.50%. If that were to increase by 26%, the prevalence would be 0.63%. In most of eastern and southern Africa, HIV prevalence is five to 25 percentage points higher than the non-African average, not 0.13 percentage points higher. Even that additional 26% overstates the impact of concurrency because it depends on assuming both the completely unrealistic transmission rate and daily sex.

After finding that actual rates of concurrency produce almost no effect on the rate of growth in HIV infections, Morris and Kretzschmar [26] try other simulations with higher, hypothetical rates of concurrency and larger numbers of partnerships than found in the Rakai survey (thus confounding the two factors). They state without citation on page 111 that "previous studies suggest that both the number of partnerships and the concurrency levels may have been higher at the start of the epidemic" [26]. None of their hypothetical simulations can produce more than a seven-fold increase in HIV infections when comparing concurrency with serial monogamy, not the 10-fold ratio that Mah and Halperin incorrectly report is based on "data from Uganda" [21]. Furthermore, that hypothetical seven-fold higher number of HIV infections requires concurrency to have fallen by nearly 80% in the period leading up to the 1994 study in Rakai and the number of partnerships to have fallen by a third (Table Three, page 118, and Figure Three, page 127, comparing scenarios No. 1 and No. 17 in reference [26]). It also requires the same unrealistic assumptions about the per-act transmission rate and the frequency of sex that make Morris and Kretzschmar's results unusable for guiding HIV-prevention policy.

Even if concurrency rates in the hypothetical past were double the actual rates found in Rakai in 1994 (and the number of partnerships was held constant), there would have been only about 75% more HIV infections after 1825 iterations compared with only serial monogamy, not the 26% more using the actual concurrency rates (Figure Three, page 127, comparing scenarios No. 8 and 9 with No. 1 in reference [26]). The evidence is mixed about whether some countries in Africa have seen significant changes in sexual behaviour in the past few decades, but there is no evidence that concurrency rates in the region dropped by 80% or even by half in the period before 1994. Of course, even if concurrency rates were higher before the 1994 study in Rakai, their relevance to policy making now, decades later, is unclear.

The data in the far right column of Table 1 show that most countries in sub-Saharan Africa have rates of concurrency below the level found in Rakai in 1994. Nationwide surveys in only three African countries (Lesotho, Côte d'Ivoire and Tanzania) measure concurrency above the level found in the Rakai survey [26,46]. Subsequent surveys in two of those countries (Lesotho and Tanzania) used a more reliable questionnaire and found concurrency near or below the Rakai level [39,67]. HIV prevalence in the third country (Côte d'Ivoire) is below the average in Africa. (Those data are discussed at length in the section on quantitative evidence.) In short, Morris and Kretzschmar's hypothetical rates of concurrency and number of partnerships are far above the actual levels found in Rakai and in other locations in the region and do not reflect the African reality. It is illegitimate for the proponents of the concurrency hypothesis to report only the most extreme results of Morris and Kretzschmar's model using hypothetical levels of concurrency far higher than actual levels as though they were presenting evidence about epidemics of HIV in sub-Saharan Africa.

**Table 1:** Concurrency rates for adult men and women from national studies reported by Halperin, Epstein, Morris-Kretzschmar and Mah, ranked by male concurrency

		GPA Surveys		DHS Surveys		Other Surveys		
Source	Country	Men	Women	Men	Women	Men	Women	Mean*
Caraël, 1995 [46]	Lesotho	55	39					47.0
Caraël, 1995 [46]	Côte d'Ivoire	36	NA					
Caraël, 1995 [46]	Lusaka	22	11					16.5
Caraël, 1995 [46]	Tanzania	18	9					13.5
Caraël *et al*, 2001 [94]	Kampala							12.0
Mishra *et al*, 2009 [39]	Haiti			16.3	0.8			8.55
Mishra *et al*, 2009 [39]	Lesotho			14.1	NA			
Morris, Kretzschmar, 2000 [26]	Rakai, Uganda			14.0	1.3			7.65
Caraël, 1995 [46]	Kenya	13	NA					
Mishra *et al*, 2009 [39]	Mali			12.0	0.6			6.30
Mishra *et al*, 2009 [39]	Guinea			11.9	1.0			6.45
Mishra *et al*, 2009 [39]	Cameroon			11.7	3.2			7.45
Mishra *et al*, 2009 [39]	Niger			11.1	0.5			5.80
Adimora *et al*, 2007, 2002 [71,72]	USA					11.0	12.0**	
Leridon *et al*, 1998 [93]	Europe-6 countries					10.0	3.1	6.55
Mishra *et al*, 2009 [39]	Swaziland			8.5	0.7			4.60
Mishra *et al*, 2009 [39]	Senegal			8.3	NA			
Caraël, 1995 [46]	Rio de Janeiro	7	0.4					3.70
Mishra *et al*, 2009 [39]	Zimbabwe			5.8	0.5			3.15
Kapiga, Lugalla, 2002 [67]	Tanzania			5.7	0.8			3.25
Mishra *et al*, 2009 [39]	Cambodia			3.5	0.2			1.85
Caraël, 1995 [46]	Manila,	3.0	3.0					3.00
Caraël, 1995 [46]	Thailand	3.0	0.2					1.60
Mishra *et al*, 2009 [39]	Rwanda			2.2	0.2			1.20
Caraël, 1995 [46]	Sri Lanka	2.0	1.0					1.50
Caraël, 1995 [46]	Singapore	2.0	0.2					1.10
Mishra *et al*, 2009 [39]	Ethiopia			1.8	0.1			0.95
Mishra *et al*, 2009 [39]	India			1.0	0.1			1.05

*Unweighted mean of men and women's concurrency

**Previous 5 years

NA = not ascertained

Definitions: Caraël, 1995 [46] and Caraël *et al*, 2001 [94] asked about "regular" partners of one year or longer with whom respondent expected sex in the future; Mishra and Bignami-Van Assche, 2009 [39] Leridon *et al*, 2998 [93] Adimora *et al*, 2002 [71] Adimora *et al*, 2007 [72] and the Rakai survey used by Morris and Kretzschmar, 2000 [26] asked respondents beginning and ending dates of partnerships to determine overlap and did not exclude short-term partnerships; Kapiga and Lugalla, 2002 [67] asked about "regular" partners of one year or longer, not otherwise defined.

### Omission of other risky sexual behaviours

Our final criticism of Morris and Kretzschmar [24-26] is that they do not model an appropriate alternative to long-term, overlapping partnerships. They assume that the only way for anyone to be unfaithful to a regular partner is to have another *long-term *partnership. In their first paper in 1996, they discuss extending their model to include both "steady and casual" partnerships, but they never do so [24]. Halperin and Epstein note that "casual and commercial sexual encounters ... occur everywhere" [20]. Those casual sexual encounters, however, are not permitted in the model that Halperin and Epstein use to support the concurrency hypothesis.

Morris and Kretzschmar model serial monogamy as an exclusive partnership that endures, on average, for months or years, followed by a hiatus without sexual contact that could also last for an extended period, followed in turn by another long-term, exclusive relationship. In the model, the only way for someone with only serially monogamous partnerships to become infected with HIV is to initiate a long-term partnership with someone already infected. It would be difficult to imagine another form of multiple partnering that carries a smaller risk of HIV infection. The model presents an exaggerated comparison, not just because it mischaracterizes long-term overlapping partnerships in a way that overstates their dangers, but also because it mischaracterizes serial partnerships in a way that *understates *their dangers by ignoring casual encounters that occur in some of those partnerships. Modelling ongoing partnerships in which there is a possibility of "casual and commercial sexual encounters" would reduce the contrast in HIV infections between concurrency and non-concurrency.

### Summary of problems with modelling concurrency

Morris and Kretzschmar have established in principle that concurrency could produce more HIV infections than serial monogamy. To do so, they had to assume daily sex with every partner, gender symmetry, unrealistically high levels of concurrency, and the absence of short-term partnerships. What the model actually demonstrates, therefore, is that concurrency cannot play an important role in African HIV epidemics.

## Quantitative evidence

In this section, we review the evidence that Halperin, Epstein and Mah present in support of the assertion that concurrency is more common in sub-Saharan Africa than elsewhere [19-23,27]. We start with the cited studies that are either not relevant to the concurrency hypothesis or otherwise provide no support, and we end with the most relevant studies. As we shall see, the relevant data undermine rather than support the notion that concurrency is more prevalent in sub-Saharan Africa than elsewhere.

### Definition of concurrency

As Lurie and Rosenthal [31] point out, the literature uses a wide variety of definitions of concurrency that produce very different measured rates, making comparisons among different studies difficult or impossible. A careful evaluation of the quantitative evidence offered by proponents of the concurrency hypothesis, therefore, must begin with a precise definition of concurrency.

Survey researchers measure overlapping partnerships in different ways. Some researchers use a broad definition of concurrency that includes one-time sexual encounters. Halperin, Mah, Epstein, Morris and Kretzschmar, however, use a narrow definition of concurrency that includes only long-term partnerships. Mah and Halperin (page 12 in reference [21]) define concurrency as "the overlap of one or more sexual partnerships for a period of one month or longer". Halperin and Epstein speak of "concurrent partnerships [in Africa] that can overlap for months or years" [19,20]. In one short article, Epstein uses the phrase "long-term" or "longer term" 17 times to describe concurrent partnerships, which she distinguishes from casual sexual contacts [23]. Mah and Halperin [27] recommend using the "consensus definition" of concurrency of the UNAIDS Reference Group on Estimates, Modelling and Projections (page 7 in reference [68]) as "multiple sustained overlapping partnerships" in contrast to merely "single long-term partnership[s] with occasional once-off sexual encounters" [68]. Morris and Kretzschmar's [24-26] mathematical model that Halperin, Epstein and Mah [19-23] use to support the concurrency hypothesis assumes partnerships that, on average, last for months or years.

Halperin, Epstein and Mah insist on defining concurrent partnerships as long-term because of the elevated transmission risk during acute infection. Mah and Halperin [21] say, "The one month time period accounts for the approximate time duration of acute HIV infection, which is an important element for transmission during concurrent partnerships." Halperin and Epstein (page 5 in reference [19]) say, "The effect of ... concurrency on the spread of HIV is exacerbated by the fact that viral load, and thus infectivity, is much higher during the initial weeks or months after infection." Halperin and Epstein repeat the argument about "the acute infection window period (typically about 3 weeks long)" and the "combined effects of sexual networking and the acute infection spike" [20]. Epstein explains that concurrency is especially likely to produce HIV transmission "because it guarantees another partner will be available during the short peak infection window" [33], which in another publication she calls the "viremic window" [23].

Infected individuals in those first few weeks must have sex frequently enough so that a significant fraction of their partners become infected. Occasional, one-time sexual encounters will not lead to a sufficient number of sex acts during acute infection to produce the rapid spread of HIV. (That also explains why the assumption of frequent sex is needed to generate the rapid spread of HIV in Morris and Kretzschmar's model.) For the concurrency hypothesis to work, partnerships must be long term.

Mah and Halperin [21] use their narrow definition of concurrency to dismiss the relevance of the "five city" study by Lagarde *et al *[38] (which found no correlation between HIV and concurrency) because "some of the concurrent partnerships may have been commercial or casual sex encounters" [21]. Nevertheless, to support their argument, the proponents of the concurrency hypothesis [19-23,27] offer numerous studies that do not measure concurrency by any definition (overlap of partnerships cannot be ascertained) or measure it only with the broad definition that does not exclude one-time contacts. Since narrowly defined concurrent partnerships are a subset of broadly defined concurrent partnerships, concurrency broadly defined is only properly used in support of the concurrency hypothesis as an upper-bound measure of concurrency. Halperin, Epstein and Mah, however, report narrowly defined and broadly defined concurrency as though they measured the same thing. Moreover, broadly defined concurrency is always higher, often much higher than narrowly defined concurrency, so the data they present often give the impression that concurrency is far higher than it actually is.

In what follows, we examine the studies that Halperin, Epstein and Mah [19-23,27,69] offer in support of the concurrency hypothesis to see if they show that long-term overlapping partnerships are more common in sub-Saharan Africa than elsewhere. The studies they cite present data for a jumble of different groups that vary by marital status or age: these groups have very different patterns of sexual behaviour. Thus, the data from the different studies cannot be compared and cannot be used to support the hypothesis.

Rates of concurrency that do not use the same numerator and denominator are also not comparable, but studies that Halperin, Epstein and Mah cite use different numerators (partnerships reported at the time of the survey, partnerships over the previous month, year or five years, and partnerships over the respondent's lifetime) and different denominators (all respondents, those who have ever had sex, and those who had sex in the previous year). Where possible, we have attempted to standardize the data by calculating concurrency as a percentage of all respondents, not just those who are sexually experienced or sexually active, and for all adults, not disaggregated by marital status or age. In doing so, we follow the recommendation of the UNAIDS Reference Group on Estimates, Modelling and Projections (page 7 in reference [68]). Where the data were insufficient to make those calculations, we note their lack of comparability.

We analyze each of the works cited as evidence by the concurrency hypothesis proponents. The criticism we offer is not of those works, but of their use as support for the concurrency hypothesis.

### Irrelevant or unusable data presented as evidence

Halperin, Epstein and Mah [19-23,27,69] present evidence as though it supports the concurrency hypothesis when it does not. These studies are irrelevant or unusable as support for the hypothesis for a variety of reasons. One of those reasons is the inability to ascertain whether the partnerships reported in the study were long term, in which case they are not concurrent as defined by Halperin, Epstein, Morris and Mah [19-27]. Some studies report partnerships that cannot even be identified as overlapping, in which case they fit neither the narrow nor broad definition of concurrency.

*Studies 1-3: *Mah and Halperin [21] refer to studies of concurrency among African-Americans to support their argument about concurrency in Africa. They also incorrectly report concurrency among African-Americans from a study by Adimora *et al *[70]. They say, "The prevalence of concurrency in the African-American population over the previous five years was 53% and 31% among men and women." Those data, however, come from a study in rural North Carolina, in which only 226 people out of a sample of 1063 could be interviewed [70]. Concurrency rates from a rural part of one state based on a small study, subject to severe selection bias, tell us nothing about "the prevalence of concurrency in the African-American population" across the United States. Moreover, Mah and Halperin inflate the reported concurrency rates by presenting data for the previous five years. They could have reported one-year concurrency rates (given by Adimora *et al *in the sentence following the one Mah and Halperin did cite) and those rates would be comparable to other studies cited by Mah and Halperin. Additionally, Adimora *et al *do not make clear if they use a narrow definition of concurrency that excludes one-time or short duration partnerships.

Mah and Halperin then report the rates of concurrency in the United States disaggregated by race from another study by Adimora and her colleagues [71]. They do not report other data from that study that are clearly relevant to a comparison among countries or world regions: that 12% of all women in the United States had a concurrent partner in the previous five years (that study did not report one-year rates) [71]. The most salient number that Mah and Halperin give in the paragraph is that 11% of all men in the United States had a concurrent partner, broadly defined, in the previous year [72]. In contrast, the most comprehensive study of African concurrency reports one-year concurrency rates (broadly defined) in sub-Saharan Africa of 8.7% for men and 0.9% for women [39].

*Study 4: *Mah and Halperin [21] incorrectly report data from a study by Gras *et al *[73] about sexual behaviour of male immigrants to Amsterdam. Mah and Halperin say that the study reports 45% of the immigrant men had concurrent partners, but that was concurrency broadly defined, not as Mah and Halperin define it. Respondents were not randomly selected from a defined population, but were "mainly recruited on the streets" and "were asked to bring their friends and family" (page 1954 in reference [73]). Although about 40% of these men did come from Africa, Mah and Halperin offer no explanation for how information about migrants to Europe is informative about sexual behaviour in Africa.

*Study 5: *Mah and Halperin [27] incorrectly report on a presentation by Campbell *et al *[74], saying that their study of heterosexual couples from seven sub-Saharan African countries "found that over a quarter of HIV seroconversions resulted from a partner outside the union". The study only says that "more than one-fourth of transmissions were unlinked to the enrolled partner" (through genetic sequencing), but offers no information about the source of unlinked transmissions. Neither Campbell *et al *nor other articles reporting on the same study describe any effort to locate outside partners to determine if they were the source of infection [75,76]. The infections could have come from prenatal examinations, tattooing, recreational drug use or circumcisions. If the infection did come from an outside sexual partner, that is evidence only of concurrency broadly defined since the study did not ask participants about the length of outside partnerships [75,76]. Mah and Halperin say, "It is not absolutely certain that these were all or mostly concurrent partnerships." There is, however, no evidence that any of the unlinked infections came from a sexual partnership, concurrent or otherwise. Moreover, couples were recruited for the study because one of the pair was infected with HIV, so the study cannot be used to say anything about broad or narrow concurrency in the population as a whole.

*Studies 6-7: *Mah and Halperin [27] report that in a Ugandan study, "the strongest behavioral association with incident HIV infection was the number of times in the past 6 months that the individual had sex with someone believed to be having sex with others" [77]. Mah and Halperin also mention a study of Zimbabwean women that found a similar correlation [78]. Those correlations cannot provide support for the concurrency hypothesis. That people infected with HIV think their partners have another partner is not evidence of anything except that people believe what they have been told. In sub-Saharan Africa, donors and governments alike relentlessly focus on a single presumed cause of transmission: risky sex. If a person has HIV and thinks the only way to get it is through sexual contact, then that person has to believe his or her partner has other partners.

*Study 8: *Halperin and Epstein [20] mention without citation the "Nelson Mandela serosurvey" in 2005 of South Africa, apparently referring to the "South African National HIV Prevalence, HIV Incidence, Behaviour and Communication Survey, 2005" commissioned by the Nelson Mandela Foundation [49]. Halperin and Epstein incorrectly report the data, saying that 40% of males aged 15-24 and 25% of females of that age "reported having more than one current sexual partner". Those numbers (from Table 3.25, page 57, in reference [49]) are only for respondents who were sexually experienced. The report never defines "current sexual partnerships" or gives the question on which those data are based. The previous table, however, reports that among those who had sex in the previous year, 27.2% of men aged 15-24 and 6.0% women aged 15-24 had more than one (not necessarily overlapping) partner in the previous year (Table 3.24, page 56, in reference [49]). That was 10.6% of all men aged 15-24 in the survey and 2.7% of all women aged 15-24. Therefore, fewer than 10.6% of men and 2.7% of women of that age were in concurrent partnerships, however defined. Accordingly, the two tables cannot both be correct. Data that are implausible because they lack internal consistency cannot provide support for the concurrency hypothesis.

On the same page as the data on current sexual partners are presented, the study reports a salient finding not mentioned by Halperin and Epstein, that the difference in HIV prevalence between respondents who had one partner in the previous year and those who had more than one partner was not statistically significant (Table 3.26, page 57, in reference [49]).

*Studies 9-11: *Mah and Halperin [27] report three studies that show concurrency plays an important role in transmission of *Chlamydia*, gonorrhea, *Trichomonas*, and syphilis in the United States [79-81]. None of the studies discriminated between concurrency broadly and narrowly defined. The importance of concurrency broadly defined in the transmission of those diseases is not evidence that concurrency, however defined, plays an important role in HIV transmission until it can be established that the transmission dynamics of those diseases and HIV are the same. Lagarde *et al*'s four-city study showed that various measures of concurrency correlate with prevalence of STIs, but not with HIV prevalence [29].

*Study 12: *Mah and Halperin [21] present data from a study by Colvin *et al *of 259 persons living in rural KwaZulu-Natal, South Africa [82]. The individuals studied were not a representative sample of any population. The researchers asked about the number of partners in the previous three months without determining (as Mah and Halperin point out) whether any of those relationships were overlapping, and thus it cannot not be ascertained if the data measured concurrency, however defined. Mah and Halperin do not report that fewer than 2% of all women in the study had more than one partner.

*Study 13: *Mah and Halperin [21] mention a study in a rural area of Swaziland by James and Matikanya, "Protective Factors: A Case Study of Ngudzeni ADP Swaziland". The study could not be located through all the standard search techniques, and so Mah and Halperin's reporting of the study cannot be verified. At any rate, Mah and Halperin state that there is nothing in the study to indicate that the reported multiple partnerships were overlapping so they do not fit any definition of concurrency. They say that the high prevalence of multiple partnering "*suggests*" that a high proportion of Swazis "are *probably *[emphasis added] engaged in concurrent partnerships" [21].

*Study 14: *Halperin and Epstein (on page 20 in reference [20]) incorrectly report data from a "national Reproductive Health Survey" in Lesotho [51]. They say it shows that "20% of men and nearly 10% of women reported having two or more partners during the past four weeks", but they report the data incorrectly. Table 3.17 in reference [51] gives the numbers as 19.3% of men and 6.1% of women, and those percentages are for only those who had sex in the previous four weeks. Only 5.6% of adult men (not 20%) and 1.8% of adult women (not 10%) had more than one partner in the previous four weeks. The study did not ascertain whether any of those partnerships were overlapping, and so they fit neither the broad nor narrow definition of concurrency. Since concurrent partnerships are a subset of all partnerships, concurrency, however defined, must have been less than 5.6% for men and 1.8% for women. The citation that Halperin and Epstein provide is a PowerPoint presentation by Halperin that contains no mention of Lesotho [83], but we were able to obtain the report of the survey by contacting the Lesotho Bureau of Statistics.

*Studies 15-16: *Epstein [23] mentions studies of South Africa by Parker *et al *and of Namibia by Parker and Connolly [84,85]. The two studies report on those who had two or more partners in the last year and in the last month, but do not report on whether those partnerships were overlapping, and thus the studies did not measure concurrency however it is defined.

*Studies 17-18: *Halperin and Epstein [19,20], Epstein [22], and Mah and Halperin [27] report information on concurrency in Thailand, Uganda and the United States. Halperin and Epstein (page 20 in reference [20]) cite Morris and Kretzschmar 1997 [25] as one source for the concurrency data, but that article contains no data on concurrency from any country. Epstein provides no citation for Morris's data on Thailand and Uganda [22]. Halperin and Epstein [19,20] and Mah and Halperin [27] cite two presentations by Morris, but standard search tools, as well as emailing the author and using the services of a research librarian, could not locate the referenced sources. After months of looking for the two presentations, a chance encounter with Morris at a conference in July 2010 led to her sending us a copy of one of the presentations, but it provided no sources for the concurrency data from the three countries and she did not respond to our email asking about her sources. Morris reported in a personal communication that she has not yet published her data from Uganda gathered in the 1990s. Therefore, none of the information on Ugandan, Thai and US concurrency can be verified.

*Study 19: *Mah and Halperin [27] incorrectly report data from a study of Botswana by Gourvenec *et al *in several ways [53]. Mah and Halperin say, "29% of individuals reported concurrent partnerships, using a composite of several definitions of concurrency." The study included only those aged 15-34, who are not representative of all adults, and the 29% was only for sexually experienced respondents. That 29%, however, measures neither narrow nor broad concurrency. The element of that "composite definition of concurrency" that was about overlapping relationships asked the respondent if he or she was still having sex or expected to have sex with more than one of his or her last three partners. Sixteen percent of sexually experienced respondents said yes, which was 12% of all respondents (Table Six in reference [53]). Since 69% of those partnerships had started within the previous month, that 12% measures only broad concurrency. Finding that only 12% of those aged 15-34 had concurrent partners, broadly defined, and that those concurrent partnerships were defined by expectations about the future, rather than actions in the past, does not provide support for the concurrency hypothesis. (A copy of the report could be found only by emailing an author.)

### Quantitative evidence from sub-national populations

Halperin, Epstein and Mah offer, as evidence for the concurrency hypothesis, several studies of sub-national populations. Finding that a city or a district has a high level of concurrency is not evidence that concurrency is high in the national population or in eastern and southern Africa as a whole, which is what the concurrency hypothesis proponents must show. Sub-national surveys are no more than a "proof of concept" that may have been useful two decades ago when the concurrency hypothesis was first suggested, but at this stage of the debate cannot serve as evidence that concurrency is substantially higher in many African countries than in other regions. Furthermore, some researchers chose to study the city or district because they thought its population was especially likely to engage in risky sexual behaviour, and thus it was chosen because it was *not *representative of the country or sub-Saharan Africa.

*Study 20: *Mah and Halperin [21] incorrectly report data from a study of one district in Bangladesh, saying that it finds concurrency among married men was 5% [86]. The actual rate of concurrency reported in the study was 6% (page 1109 in reference [86]). The sample used in the study was not randomly drawn, and the article does not clarify how respondents were recruited.

*Study 21: *Mah and Halperin [21] incorrectly report data from Voeten *et al*'s study of Kisumu, Kenya, and nearby rural districts in two ways [87]. First, the concurrency rates in the study are for sexually active men and women aged 15-29, not everyone of that age, as Mah and Halperin report. Second, Voeten *et al *say that concurrency among married males in rural areas was 21% (page 484 in reference [87]), not the 27% that Mah and Halperin incorrectly report. The data in the study are for "concurrency at the time of the interview", but how relationships were determined to be concurrent was not explained. The authors of the study state that the risky sexual behaviours that they found in Kisumu were unusually prevalent "compared with DHS surveys from 28 sub-Saharan African countries" [87], so Voeten *et al *find that Kisumu does not reflect sexual behaviour in sub-Saharan Africa as a whole.

*Study 22: *Mah and Halperin [21] report on another study of Kisumu by Mattson *et al *that measures concurrency over the respondent's lifetime among sexually active men aged 18-24 [44]. Concurrency is, by definition, higher among sexually active men than among all men, is higher over a lifetime compared with the previous year or at the time of the interview for a population, and is reportedly higher for youth than all adults. Thus the concurrency rates in the study cannot be compared with those from any other study that Mah and Halperin cite. The study does not make clear whether or not it uses a narrow definition of concurrency that excludes one-time or short-duration partnerships. The authors of the Kisumu study make no claim that respondents were a representative sample of Kenyans or even of sexually active men in Kisumu.

Mah and Halperin do not report that, after adjusting for other factors in a multivariate analysis, Mattson *et al *[44] found no statistically significant association between concurrency and HIV prevalence among respondents in the study. Mah and Halperin also do not report that the behaviours that the study did find to be statistically associated with HIV infection were recent tattooing, receiving injections, bloodletting and having a history of sexually transmitted infection (pages 3, 4 in reference [44]).

*Study 23: *Mah and Halperin [27] cite a study in a rural district in South Africa by Harrison *et al *of youth aged 15 to 24 [47]. Among sexually active men, 38% reported a second regular partner at the time of the interview, but that was 23% of all male respondents (page 298, 301 in reference [47]). Mah and Halperin do not report that only 1% of women in the study had a concurrent partner.

*Study 24: *Mah [69] reports on a study in Western Cape Province of South Africa of young sexually experienced adults aged 16 to 26 years. Respondents were asked if they "had sex with a concurrent partner while in the most recent sexual partnership" (page 105 in reference [69]). Without any information about what the respondent thought a concurrent partner was (even the experts cannot agree), one cannot know what the question measured. Among the sexually experienced, "concurrency" was 12.8%. The study reports two different numbers for the sexually experienced, so "concurrency" for the whole sample was either 7.7% or 9.0%. That prevalence of concurrency for young adults is low by international standards.

*Studies 25-26: *Mah and Halperin [21] incorrectly report evidence from the Carter *et al *[88] study of Botswana. They say that "23% of the sexually active respondents reported having had a concurrent partnership with any of their last three partners over the last 12 months". Those 23% reported on line 1 of Table Two (in reference [88]) were not necessarily in concurrent partnerships, as Mah and Halperin define concurrency, since they included partnerships that were not long term. On line 2 of that table, the study does report concurrency as Mah and Halperin use the term: 7.7% of sexually active respondents were "in two or more concurrent partnerships at the time of the survey", which was only 4.2% of all respondents (34 out of 807). The 23% of sexually active respondents who had broadly defined concurrent partnerships were 16% of the whole sample. Those percentages would be still lower, perhaps much lower, if the overlap of concurrent partnerships had been measured the standard way, by asking about overlap in the previous year, rather than since the beginning of the partnership (which could have been years before the interview). Moreover, the survey was conducted in only seven of 24 health districts in the country and thus is "not strictly generalizable to the national level" [89].

After discussing Carter *et al *[88], Mah and Halperin [21] immediately add that "another survey in 2003 in Botswana found a similar prevalence of concurrent partnerships" and cite Meyerson *et al *[89]. The Meyerson *et al *study, however, reports on the same survey (the 2003 Makgabaneng Radio Serial Drama Listenership Survey) as Carter *et al*, not on another survey.

In sum, the proponents of the concurrency hypothesis cite seven studies not already discussed from which they report concurrency rates in sub-national populations. At least two of the studies are of areas with risky sexual behaviour well above the norm for sub-Saharan Africa, and so cannot be portrayed as representative of sub-Saharan Africa as a whole. Even though the concurrency hypothesis is necessarily comparative, only one of these studies is of a non-African country, and that study gives concurrency rates only for married men, a group that reportedly has lower concurrency rates than all adult men. Four of the African studies report data for young adults who reportedly have higher rates of concurrency than all adults. Hence, these five studies exaggerate the difference between Africa and the rest of the world. Two of the studies reported data from the same survey.

Only one of the studies [88] reports data from which one can calculate concurrency rates, narrowly defined, for all adults in order to make the necessary comparative judgments. That study found that 4.2% of adults in eastern Botswana were in concurrent partnerships, which is low compared with non-African countries with low HIV prevalence (see Table 1), and unambiguously contradicts the concurrency hypothesis. None of the other studies of sub-national populations provides unambiguous support for the hypothesis.

### Other quantitative evidence

Mah and Halperin [21] cite Wellings *et al*, who contend that concurrency may be common in parts of Africa [5]. Wellings *et al *is a comprehensive review of surveys of sexual behaviour from 59 countries. Since few of those surveys asked questions about concurrency, the article presents no data on the subject. Nevertheless, Wellings *et al *say, "Evidence is available that, although lifetime number of partners might be lower, concurrent partnerships in men in some African countries might have been more common and of longer duration than in other regions" (page 1957 in reference [5]). That assertion by Wellings *et al *references five studies:

*Study 27: *Mwaluko *et al *conducted a survey in a rural district in Tanzania [90]. The study reports no data on concurrent partnerships, however one might define concurrency. Instead, the paper presents data on the number of partners in the previous year and median age at first sex.

*Study 28: *Kapiga and Lugalla present an analysis of the 1996 DHS survey of Tanzania [67]. Concurrency, narrowly defined, was 5.8% among men and 0.8% among women. Those data are, in fact, evidence that African concurrency is not "more common ... than in other regions" [5]. (See Appendix D for calculations of the concurrency rates.)

*Study 29: *Ferguson *et al *studied 252 men in four groups of informal sector workers recruited to join an HIV-education programme in a small town in Kenya, and thus were not a representative sample of any population [91]. The study reports on potentially risky sexual behaviour, including the number of partners in the previous year, but not whether any of those partnerships overlapped. The only data on concurrency in the study are that two men reported two wives and no additional partners, and 7% of the men reported a wife in addition to a regular partner. Altogether, 8% of respondents reported concurrent partnerships, narrowly defined (page 436 in reference [91]). Those data are not evidence that concurrency is common in Kenya, especially so because the respondents were chosen apparently because of a presumed high occupational risk of engaging in risky sexual behaviour.

*Study 30: *Williams *et al *is a study of South African miners, commercial sex workers, and people living near the mines [92]. The study reports no data on concurrent partnerships, but instead reports on the number of lifetime partners and "on-going casual partnerships", for which no definition is given. It is likely that the community was selected for study because of its presumed high prevalence of risky sexual behaviour.

*Study 31: *Leridon *et al *present data for six countries in western Europe in which respondents were asked about concurrent partnerships, broadly defined [93]. Among heterosexual adults, an average of 10.0% of European men said that they had another partner in addition to their steady partner, and 3.1% of European women did. Comparable figures in sub-Saharan Africa are 8.7% for men and 0.9% for women [39].

Thus, the five studies cited by Wellings *et al *do not support the concurrency hypothesis. Those studies include two articles that do not report any data on concurrency, however defined, two that report low levels of narrowly defined concurrency in Africa, and one study showing higher levels of broadly defined concurrency in Europe than in Africa.

### National data from the GPA and DHS

Before the 1990s, there were few published surveys on sexual behaviour in low- and middle-income countries. The WHO's Global Programme on AIDS (GPA) tried to fill that gap by commissioning seven national and four single-city surveys of sexual behaviour in 1989 and 1990 that measured concurrency (Studies 32 and 33 in Additional file 1: Table S1) [46,94]. Reported concurrency in the four surveys of African countries was substantially higher than elsewhere. Men's concurrency in the four African countries ranged from 13% to 55%, far above the 3% to 7% found in the three Asian countries surveyed. The fourth and fifth columns of Table 1 include the seven national and four city GPA surveys that measure concurrency.

The GPA surveys are the only data on concurrency presented in Halperin and Epstein (2004) [19] and Epstein [22]. In addition to the GPA surveys, Halperin and Epstein (2007) [20] mention only two other sources of concurrency data [49,83], the deficiencies of which we have discussed. Mah and Halperin [21,27] begin their presentations of empirical evidence with the GPA surveys. The foregoing pages, however, show that none of the other studies they cite provides unambiguous support for the concurrency hypothesis and many of them contradict the hypothesis. Data from the 11 GPA surveys are therefore the key empirical evidence for the concurrency hypothesis, and so an extended discussion of those surveys is warranted.

The US Agency for International Development (USAID) established the DHS and worked with a series of non-governmental agencies and Johns Hopkins University, helping to standardize and improve the quality of survey research in low- and middle-income countries. The DHS has produced 14 surveys that have measured concurrency [39,67]. Table 1 presents the DHS data in columns six and seven, ordered from highest to lowest male concurrency rates. Concurrency rates measured by the GPA and the DHS surveys barely overlap in Africa. In the sub-Saharan African countries for which the DHS measured concurrency, the average rate for men was 8.5% and the average rate for women was 0.8%.

Only in Lesotho and Tanzania are there head-to-head comparisons between the GPA and DHS surveys. The GPA found 55% male concurrency in Lesotho, but the DHS found 14.1%. The contrast was also considerable in Tanzania, where the GPA measured men and women's concurrency as 18% and 9%, respectively, but the DHS reported it to be 5.8% and 0.8%. (See Appendix D for the calculation of those concurrency rates.)

The stark difference between the GPA and DHS surveys must be addressed carefully in order to sort out whether there is any quantitative evidence that supports the concurrency hypothesis. If the GPA data are correct, then the proponents of the concurrency hypothesis have data for two cities and four countries in Africa (two of which do not measure female concurrency) to support their argument. Even if the GPA data do not reflect concurrency now, but did measure it correctly in the past, they have some, albeit slim, historical evidence, whose relevance to current policy is unclear. What follows, then, is a detailed comparison of the GPA and DHS surveys.

Mah and Halperin [27] never seriously address the DHS surveys of African concurrency. They try to dismiss the DHS surveys by saying, "the study had several major limitations," of which they mention only two. First, they state that there is "the likelihood of substantial underreporting [of concurrency] on such household surveys". Nevertheless, Mah and Halperin cite 12 studies that report on 27 household surveys as quantitative evidence in support of the concurrency hypothesis. They do not explain why the GPA and other household surveys, which they accept, are more accurate than the DHS surveys they reject. Moreover, the DHS surveys were all conducted in private (page 5 in reference [39]) and the GPA surveys were conducted in private only "wherever possible" (page 21 in reference [95]), making DHS surveys less vulnerable to underreporting than the GPA surveys [39,95]. The only practical way to conduct a nationally representative survey is to use a sampling frame of households, and if one samples households, then the interview will almost inevitably take place in the home. Mah and Halperin's criticism of the DHS is invalidated by their own reliance on household surveys for evidence to support the hypothesis.

The only other reason that Mah and Halperin [27] give for rejecting the DHS surveys is that some of the surveys did not gather information on the "duration of the respondents' sexual partnerships with the second-to-last or third-to-last partners" [27,39]. That is true only for one African country, Ethiopia, where respondents were asked about only two (the current and previous), instead of the four most recent partnerships. Mah and Halperin give no valid reason for dismissing the evidence from the other nine African countries surveyed by the DHS.

Morris *et al *[96] argue that defects in the way the DHS asks questions about sexual partnering lead to potential underestimates of rates of concurrency. Until their paper is published, their criticism cannot be evaluated, but it should be noted that, of the countries with possible measurement error listed in the abstract of their conference presentation, only two (Swaziland and Zambia) were countries included in Mishra and Bignami-Van Assche's article [39] that we use as our source of DHS measures of concurrency. Morris *et al*'s abstract does not indicate the years of the surveys, so it cannot be determined that even the Swaziland and Zambia surveys that they criticize are ones included in Mishra and Bignami-Van Assche's study.

The DHS and GPA surveys use different definitions of concurrency. The GPA surveys and the DHS survey in Tanzania use a narrow definition of concurrency: they ask about regular partnerships that last a year or longer [67,95]. The other DHS surveys, in contrast, use a broad definition of concurrency; their definition of overlapping partnerships could include one-time encounters [39]. Nevertheless, the DHS data (other than in Tanzania) are useful in this instance because they provide an *upper-bound *measure of concurrency, as defined by the concurrency hypothesis, since narrowly defined concurrent partnerships are a subset of broadly defined ones. All but one of the DHS surveys in Africa report concurrency lower than in any of the GPA surveys from Africa, and those DHS surveys overstate concurrency, narrowly defined. Furthermore, the GPA surveys and the DHS survey in Tanzania measured concurrency at the time of the interview [46,67]. The other DHS surveys measured concurrency in the previous year, which has to produce the same or higher concurrency rates than concurrency measured at the time of the interview [39]. Accounting for those differences in the definitions of concurrency used by the GPA and DHS widens the discrepancy between the two sets of surveys rather than helping to reconcile them.

One explanation for the disparity between the two sets of surveys is that they both accurately measured sexual behaviour, but that concurrency rates fell during the period between the two sets of surveys. The fieldwork for the GPA surveys was conducted in 1989 and 1990 [95]. The DHS survey in Tanzania was carried out in 1996 and the other DHS surveys were conducted between 2002 and 2006 [39,67]. Some argue that people in sub-Saharan Africa have changed their sexual behaviour in response to the epidemic [97-99]. Nevertheless, if both the GPA and DHS surveys are correct, there would have been a precipitous decline in concurrency in Tanzania in the first half of the 1990s (men's concurrency rates would have fallen by over two-thirds, and women's by over 90%), but other survey data in Tanzania show that sexual behaviour hardly changed in the 1990s. In reporting the 1996 survey, Kapiga and Lugalla (page 461 in reference [67]) say that only 7% of respondents reported *any *reduction in risky sexual behaviour after learning about AIDS. Mwaluko *et al *also report a "striking lack of change in sexual behaviour" in Tanzania between 1995 and 2000 in a study that did not specifically examine concurrency (page 2645 in reference [90]). It is even less plausible that male concurrency in Lesotho could have fallen from 55% to 14% in 15 years.

The most likely explanation for the very different reported rates of concurrency in Africa in the GPA and DHS surveys has to do with differences in the questionnaires. The UNAIDS Reference Group on Estimates, Modelling and Projections [68] warns against measuring concurrency by asking respondents if they had regular partnerships at the time of the interview and whether they expected the partnership to continue (page 10 in reference [68]). Instead, the Reference Group report says that respondents should be asked to specify the beginning and ending date of each partnership. The objective is to avoid "culturally defined notions of relationships" and, of course, mere wishful thinking.

Respondents in the GPA surveys were asked if they had a "regular" partner, defined as someone with whom "you intend to continue having sex" [95]. In contrast, the definition of concurrency used in the DHS surveys after 2000 used wording that was close to the UNAIDS recommendation [39]. In some DHS surveys, respondents were asked to give the date of the first and last sexual contact for their most recent partner, and in other surveys, respondents were asked the dates of the second- and/or third-to-last partnerships. Partnerships were defined by those dates, not by a culturally constructed conception of the meaning of "regular partnership". None of the DHS surveys asked about hoped-for sex. The GPA and DHS questionnaires embed different definitions of sexual partnerships that likely explain much or all of the difference in measured concurrency.

Even if the GPA accurately measured concurrency in 1989 or 1990, it is not clear what the very high rates in Lesotho and Tanzania tell us about eastern and southern Africa as a whole. Lesotho, with the highest measured concurrency, does have one of the worst HIV epidemics in Africa and that could be considered evidence in support of the concurrency hypothesis. Nevertheless, Caraël reports that Lesotho - a country completely surrounded by South Africa - has "temporary migration of more than half of the adult male population ... that leaves couples separated for most of the year" [46]. The migrant has a long-term relationship with the spouse left behind, but that cannot entail frequent sex. That is not the kind of concurrency modelled by Morris and Kretzschmar that assumes daily sex. Moreover, the DHS survey puts men's concurrency in the country at 14.1%.

As Lurie and Rosenthal point out [32], Côte d'Ivoire, the country in the GPA surveys with the second highest concurrency rates, has HIV prevalence well below the average for sub-Saharan Africa, so that contradicts the concurrency hypothesis. Tanzania, the country with the third highest concurrency in the GPA surveys, as noted, was subsequently resurveyed only six years later, finding very much lower concurrency. In Kenya, the country with the fourth highest concurrency in the GPA surveys, men's concurrency was 13%, slightly higher than the 11% in the United States [72]. The two other surveys by the GPA were in Lusaka and Kampala; we have offered reasons why one should discount sub-national surveys. If those six surveys were the only data available, one might imagine that the concurrency hypothesis *could *be true if only more data would confirm the findings of those first few surveys. But many more surveys have been conducted and not one of them confirms what was found in the GPA surveys.

### Summary of the quantitative evidence for the hypothesis

In summary, the proponents of the concurrency hypothesis say concurrency is common in Africa, but "the pattern of serial monogamy [is] more common in the West" [20]. Nevertheless, they present hardly any data on concurrency in the West and never point out that empirical evidence shows concurrency in the West is high by global standards. They present no direct evidence on serial monogamy anywhere and so do not show that it is more common in the West than in Africa.

In addition to the 11 GPA surveys [46,94], Halperin, Epstein and Mah cite 31 other studies, only seven of which give any information about non-African countries. They incorrectly report data from 13 of the 31 studies. (See Additional file 1: Table S1 for a summary of problems with the studies.) In every instance, their error exaggerates the difference in concurrency between Africa and other regions. They report data from seven studies on non-concurrent sexual behaviour as though they were relevant to the concurrency hypothesis. Two of those seven studies also provide data on concurrency rates that they do not report. Eight other studies they cite are irrelevant to the concurrency hypothesis for other reasons.

They report data from 25 surveys of cities and rural districts that are not necessarily representative of the country in which they were located (or, in some cases, even of those cities or districts), not to mention sub-Saharan Africa as a whole. In at least five of the studies, the researchers likely selected the group or sub-national area to be studied because of its expected high prevalence of risky sexual behaviour: that is, because they were *not *representative of the population or the country in which they were located. Ten of the studies use small and/or non-random samples from which one cannot make useful statistical inferences. Only two of the 31 studies unambiguously report concurrency rates for all adults in the area studied and they show that concurrency in Africa is low by global standards. In sum, we find that of the studies they offer as evidence, none provides unambiguous support for the concurrency hypothesis and more than a few contradict the hypothesis.

Seven of the cited studies are about young adults, whose reported concurrency rates are typically higher than for all adults. Finding that many African young people have concurrent partners is not evidence for the concurrency hypothesis unless the African data are compared with non-African data, but the hypothesis proponents do not offer any evidence about youth concurrency from outside Africa. Mah and Halperin [27] cite an article by Drumright *et al *about concurrency and STIs (not about HIV), but do not report what the article says about youth concurrency in the United States: "Concurrent partnerships have ranged from 32% to 54% among adolescents ... in different regions of the United States" [79]. Those percentages are higher than any rates of concurrency for youth that Halperin, Epstein and Mah found in Africa.

Our analysis of quantitative evidence offered for the concurrency hypothesis has distinguished between narrowly defined and broadly defined concurrency. We have pointed out that broadly defined concurrency is always larger than narrowly defined concurrency, making comparison between different studies difficult, and we have argued that only narrowly defined concurrency is relevant to a test of the concurrency hypothesis. Nevertheless, it is worth noting that none of the 31 studies cited by Halperin, Epstein and Mah report credible data showing that broadly defined concurrency is higher in sub-Saharan Africa than elsewhere.

The most useful data for verifying the concurrency hypothesis has to be nationally representative studies of all adults. Almost all such studies come from the GPA and the DHS, but those two sets of surveys paint dramatically different pictures of the level of concurrency in Africa. Halperin and Mah ignore or dismiss, without justification, reliable data on concurrency in Africa from the DHS surveys that suggest that Africans are less likely to be in concurrent partnerships than people in the United States or Europe. The case for the concurrency hypothesis rests largely on high rates of concurrency in GPA surveys in Lesotho and Tanzania. But in both countries, subsequent surveys found much lower levels of concurrency. It is close to inconceivable that reductions in risky sexual behaviour could explain the different concurrency rates from the GPA and DHS in Lesotho and Tanzania, but differences in the questionnaires in the two sets of surveys indicate that the DHS surveys are more reliable since they used the UNAIDS recommended questions.

Studying sexual behaviour across the world is a complicated business. One could not complain if Halperin, Epstein and Mah, in reporting on dozens of studies, made a reporting error here or there, reported some data that could not be compared with other data, cited some evidence that only suggested, but did not clearly confirm, the concurrency hypothesis. We find the frequency of errors in the reporting by Halperin, Epstein and Mah to be unacceptably high. We find that most of the studies they offer as evidence in support of the concurrency hypothesis do not provide comparable data, that most are not even relevant to a test of that hypothesis, and that none provides unambiguous support for the hypothesis. The totality of their evidence does not add up to support for the concurrency hypothesis.

## Qualitative evidence

The final category of evidence that the proponents of the concurrency hypothesis offer is what they call qualitative evidence: the attitudes, perceptions and beliefs related to concurrency [21,27]. Although the concurrency hypothesis is essentially comparative, they present no evidence about attitudes, perceptions and beliefs related to concurrency outside of Africa. Thus, none of the qualitative evidence that Halperin, Epstein and Mah [19-21,27] offer can support the concurrency hypothesis. Even if they could show that concurrency is widely accepted in Africa (we argue that they do not), but fail to show that it is not widely accepted elsewhere, they have not provided evidence for the hypothesis.

Another reason why all of their qualitative evidence is irrelevant, pointed out by Lurie and Rosenthal [31], is that generating new HIV infections depends upon what people actually do, not what people think or feel about their own concurrency or someone else's sexual behaviour.

A third flaw in Mah and Halperin's argument [21,27] has to do with standards of evidence about the prevalence of attitudes. Careful surveys about attitudes toward a particular behaviour, based on random samples of defined populations, can determine whether or not those attitudes are common in those populations. Studies whose respondents are not carefully selected to be representative of a defined population cannot produce evidence about the prevalence of perceptions or attitudes. Mah and Halperin say that "qualitative data indicating that concurrency is a highly normalized behavior in many parts of southern and eastern Africa is now rather overwhelming" [27]. They offer two citations in support of that assertion. One is based on interviews with "228 members of southern African non-government organisations representing seven countries" [100]. The study provided no information about how those 228 respondents were selected, so we do not even know if their perceptions are representative of non-governmental organization members generally, and they certainly do not speak for all southern Africans. The other is a study by the Soul City Institute that reported on 179 focus groups of about 1900 people in 10 countries in eastern and southern Africa and in-depth interviews with 116 of the participants [101]. The report states that the organizers of some focus groups specifically recruited participants from groups suspected of high-risk behaviours ("truck drivers, migrant workers, cross-border traders, and uniformed personnel") [101]. For the in-depth interviews, as Lurie and Rosenthal point out [32], the researchers in all but one country selected only people with multiple partners. Conversations with truckers and soldiers selected for having multiple partners cannot provide evidence that concurrency is "highly normalized" behaviour in sub-Saharan Africa.

Mah and Halperin [21,27] make a fourth mistake when they confuse qualitative and quantitative evidence and the relationship between the two. Qualitative evidence can reveal some kinds of important information. It can elucidate "how and why people behave, think, and make meaning" of their lives, and it falls "within the context of discovery rather than verification" [102]. Qualitative evidence about attitudes cannot reveal whether or not concurrency is common since that is a quantitative statement (even though imprecise) about people's actions. Research that investigates only perceptions and attitudes about concurrency can only reveal what people think about concurrency, not how they or other people behave.

Following discussion of their two sources on attitudes, Mah and Halperin say, "While it is true that qualitative data cannot be used to estimate the numeric prevalence of concurrency in a given population, *they do provide compelling evidence that this type of sexual partnering is common *in southern and parts of east Africa. We have yet to find any *qualitative studies examining concurrency in the region that did not find it to be common *[emphasis added]" [27]. In their view, qualitative evidence *is *quantitative evidence. Finding that many people think concurrency is common, however, does not establish that concurrency is common, and certainly cannot provide "compelling" evidence that it is.

Mah and Halperin assert that concurrency is a natural part of the African cultural landscape, saying that in sub-Saharan Africa, "historical explanations of multiple concurrent partnerships are rooted in biology and polygyny" [21]. There is no basis for claiming that Africans are biologically different from non-Africans such that they are more likely to enter into concurrent partnerships or approve of others doing so. Mah and Halperin also offer no explanation for why polygyny predisposes Africans to non-polygynous concurrency, but not people in the dozens of other countries where it is legal and common and HIV prevalence is much lower than in sub-Saharan Africa. Mah and Halperin also say that the "roots of concurrency" are found in the migrant labour system in Africa, which "resulted in men and women spending considerable time apart" [21]. African sexual partners who spend considerable time apart cannot have the frequent sex that is a necessary presumption of the concurrency hypothesis. Moreover, long distance labour migration is widespread across the globe, not just in Africa.

In their attempt to show that high rates of concurrency in Africa are plausible, the concurrency hypothesis proponents bring into the argument the assertion that transactional sex is common in sub-Saharan Africa. The literature on transactional sex suffers from the same lack of comparative perspective seen in the literature on the concurrency hypothesis. All over the world, people who have sex with each other also have other dimensions to their partnership, and some of those dimensions involve exchanges of services, goods and love, not just sex. People who bond through sex often want to give their lovers gifts to show their affection and to intertwine their lives in myriad other ways - everywhere in the world.

Halperin and Epstein [20] say, "Although most African women in concurrent partnerships are not sex workers, such relationships often include a powerful element of sexual-economic exchange ...." Morris and Kretzschmar put it this way: "Only a handful of respondents in this survey identified their partners explicitly as a prostitute (although about 80% report some kind of economic support as given or received, reflecting the difficulty in identifying 'commercial' sex in this kind of population)" [26]. Mention of transactional sex pervades the literature on sexual behaviour in Africa. For example, James Shelton of USAID writes in *The Lancet *that, "transactional sex ... arguably reflects the *norm *[*sic*] for sexual relationships [emphasis added]" in eastern and southern Africa [103].

Picture the reaction if *The Lancet *were to publish an article that said, "About 80% of US women reported receiving flowers, poetry, candy or jewellery for Valentine's Day, and such transactions in sexual relationships are the norm in the population. Some women also reported periods of financial support from a sexual partner. It is difficult to identify commercial sex in this kind of population, and so we cannot explicitly identify all 80% of US women as prostitutes."

In sum, the proponents of the concurrency hypothesis [19-23] present qualitative evidence that they believe shows that concurrency is both widely accepted and common in sub-Saharan Africa. They present no information about attitudes and perceptions outside of Africa. Their evidence cannot show that concurrency is common, it cannot show that it is more common or widely accepted in Africa than elsewhere, and it cannot even show that it is widely accepted in sub-Saharan Africa.

Their qualitative evidence turns out to be no evidence at all, but it still serves to promote the concurrency hypothesis since its effect is to portray "African" attitudes and behaviour as peculiar and anomalous. Much of their qualitative "evidence" does nothing more than evoke widely held stereotypes about Africans that have been deeply embedded in Western culture for centuries [3]. Connecting the notion of concurrency to those stereotypes thereby desensitizes the reader to the weakness or irrelevance of much of their "evidence".

## 20 years are enough

The evidence that Halperin, Epstein, Kretzschmar, Morris and Mah [19-23,26,27,34,69] assemble does not show that concurrency is a main driver of the HIV epidemics in eastern and southern Africa. To make that case, they would have to demonstrate the validity of two assertions, that concurrency leads to greater, indeed much greater, epidemic spread of HIV than other patterns of sexual behaviour, and that concurrency is more common, maybe much more common, in the region than elsewhere. They have not demonstrated the validity of either assertion.

Morris and Kretzschmar's model actually shows that concurrency does not lead to significantly faster spread of HIV compared with serial monogamy. Rather than supporting the concurrency hypothesis, the model proves that the hypothesis is invalid. Morris and Kretzschmar's [26] model could be put to good use exploring what is really driving the sub-Saharan African HIV epidemics, but not until its unrealistic parameters are replaced.

The proponents of the concurrency hypothesis would also have to demonstrate that concurrent sexual partnerships are more common in Africa than elsewhere, but none of the studies they cite provides unambiguous support for that assertion and more than a few contradict it. The proponents of the hypothesis can persist in claiming that concurrency is more prevalent in Africa than elsewhere only by ignoring much of the useful and relevant data from Africa, as well as from the United States, Europe and Latin America.

The efforts to explain the extraordinary difference in HIV prevalence between eastern and southern Africa and the rest of the world by differences in concurrency or other sexual behaviours have failed. Of course, sexual behaviour does have much to do with an individual's risk of acquiring HIV, and we do not dismiss the role of efforts to change sexual behaviour in HIV-prevention policy. But sexual behaviour alone simply cannot explain the extraordinarily high HIV prevalence in much of Africa. An epidemic, especially one of the scale and diversity of HIV, is a complex, contingent process that results from numerous, interacting factors [104].

We suggest here two possible explanations for Africa's extraordinary HIV epidemics - there is already a substantial body of research on each - only to illustrate the kinds of factors that should be considered. Once it is recognized that sexual behaviour is not driving African epidemics, it will be possible for many other possible factors to be considered.

First, per-act heterosexual transmission of HIV between otherwise healthy adults, even during acute infection, is very low, but many bacterial, viral and parasitic infections can make infected partners more infectious and uninfected partners more vulnerable over extended periods by raising transmission rates [58,59,61,105,106]. The high transmission rate assumed in Morris and Kretzschmar's model suggests that cofactor infections could accelerate the epidemic. The effect of cofactors could be explored using their model by first replacing the unrealistic assumptions and then assuming higher per-act transmission rates for a portion of the individuals, during both the acute and asymptomatic infection periods.

The role of STIs in promoting HIV transmission has been widely discussed. For example, after finding a strong association between HIV and HSV-2 in the four-city data, Auvert *et al *say that the "differences in efficiency of HIV transmission as mediated by biological factors outweigh differences in sexual behaviour in explaining the variation in rate of spread of HIV between the four cities" [107]. Additionally, urogenital schistosomiasis (*Schistosomiasis hematobium*), found mostly in Africa, produces urogenital lesions in women and men, increases viral shedding of the infected partner, and produces genital inflammation for the uninfected partner, all of which facilitate transmission of HIV [108-112]. Women with genital lesions of schistosomiasis were three times as likely to contract HIV as women in the same Zimbabwean villages who did not have those lesions [113]. Malaria also raises viral load, making the infected partner more contagious [114-116]. The burden of those and other diseases suspected of increasing HIV transmission is far higher in sub-Saharan Africa than elsewhere [117-120].

Another explanation for the African HIV epidemics that merits further attention is blood exposures, such as unsterilized syringes, other invasive medical and dental procedures, circumcision (either in a medical setting or elsewhere), treatment by informal injectionists, tattooing, sharing of hairdressing equipment, therapeutic bloodletting and so on [121-127]. Many forms of blood exposure are far more efficient at transmitting HIV than most heterosexual behaviours. Even WHO admits that 30% of injections in eastern and southern Africa use unsterilized needles and 7-12% of new HIV infections worldwide come from unsterile injections and blood transfusions [128-130]. Morris and Kretzschmar's model could also be used to explore the impact of blood exposures. New infections could be seeded as the model is iterated (not just at the beginning) to simulate their impact.

It is customary to end the presentation of research with calls for still more research. This paper, however, calls for an end (or at least a moratorium) to research on sexual behaviour in Africa of the kind discussed in this article. The continued use of financial and human resources to prove Western preconceptions about African sexuality cannot be justified.

Researchers have developed extraordinary skills and institutional structures to carry out survey research in developing countries. Instead of asking about the starting and ending dates of respondents' third-to-last sexual partnership, we need to use those research resources to ask about other possible correlates of HIV risk. What the armies of survey researchers blanketing Africa need to learn is information about, for example, use of bed nets (since malaria raises transmission rates), sanitation (since *Schistosomiasis hematobium *and possibly other parasitic diseases raise transmission rates), nutrition (since poor nutrition undermines the immune system and speeds the progression to AIDS, increasing infectivity), STIs (since they increase contagiousness and vulnerability to HIV), recreational drug use, homosexuality, and the numerous forms of blood exposures that could promote HIV transmission. These are some of the questions to which policy makers need answers in order to understand what is driving the African HIV epidemics.

Until now, the obsession over sexual behaviour - most recently, the focus on concurrency - has blocked efforts to understand the HIV epidemics in sub-Saharan Africa and to devise effective prevention programmes. A comprehensive effort to determine what is driving the African HIV epidemics is long overdue.

## Appendix A

The data that Morris and Kretzschmar [26] report from the 1994 Rakai study indicate that 20.2% of all partnerships were concurrent, but that number must be calculated from the data they include in their article (Table Two, page 112, 118 in reference [26]). Those data show 32.3% of men and 26.7% of women had no partner, 53.2% of men and 71.9% of women had one partner, 12.4% of men and 1.1% of women had two partners, and 2.0% of men and 0.2% of women had three. From those numbers, one can determine the rate of concurrency for men (12.4 + 2.0 = 14.4), the rate of concurrency for women (1.1 + 0.2 = 1.3). One can also find the proportion of partnerships that are concurrent by counting up the total number of men and women's partnerships and the number of men and women's partnerships of those with more than one partner. Weighting those results by the ratio of men to women (0.89) produces the result that 20.2% of partnerships were concurrent in Rakai.

Morris and Kretzschmar in 1997 assume gender symmetry [25]. They present a graph showing the number of new cases of HIV assuming different proportions of partnerships that are concurrent (Figure Three, page 645, in reference [25]). When 20% of partnerships are concurrent, there are about 240 infections, which is 100% more than the 120 cases with only serial monogamy. Another way to reproduce the same result is to use their statement that each time the proportion of partnerships that are concurrent is raised by 10 percentage points, the size of the epidemic grows by 40% (page 645 in reference [25]). From zero to 20% concurrency thus leads to 1.96 times as many cases, or about 100% more cases if 20.2% of partnerships are concurrent.

Morris and Kretzschmar also use data from Rakai to specify longer average partnership duration and that may also have reduced the contrast between concurrency and serial monogamy.

## Appendix B

Epstein asserts, "While the transmission rates used by Morris and Kretzschmar are high, this does not affect the comparison between concurrency and serial monogamy" [33]. It is true that a lower transmission rate in Morris and Kretzschmar's model would lead to a smaller number of new infections at every level of concurrency, including when no partnerships are concurrent, but it is not correct that the "comparison between concurrency and serial monogamy" would be unaffected by changes in the transmission rate. Epstein's error can be explained by reference to Morris and Kretzschmar's graph (Figure Three, page 645, in reference [25]), which shows the average number of HIV infections (on the vertical axis) after 1825 iterations at different levels of concurrency (on the horizontal axis). Morris and Kretzschmar [24-26] repeatedly describe this curve as exponential, but it is not exponential in a formal mathematical sense since it is not a representation of an equation in which an exponent is a variable. Instead, the graph represents hundreds of stochastic simulations. It is exponential only in an informal sense that the slope of the curve is positive and the slope rises steadily as the level of concurrency increases. Consequently, a formal mathematical proof refuting Epstein's assertion is not possible, but the following demonstrates the error in her argument.

The graph in Figure Three (in reference [25]) assumes a 0.05 transmission rate (and daily sex between all partners), as do all of Morris and Kretzschmar's calculations. The intercept of the curve - approximately 120 - is the number of infections after 1825 iterations with no concurrency. The slope of the curve reflects the difference in the number of infections with only serial monogamy compared with the number of infections at increasing levels of concurrency. For Epstein to be correct - that changing the transmission rate would not affect the comparison between concurrency and serial monogamy - any reduction in the transmission rate below 0.05 would have to leave the slope of the curve unchanged. To understand why that cannot be so, picture the curve when the transmission rate has fallen to its mathematical limit of zero. At that point, there is no transmission of HIV and the curve is a horizontal line with an intercept of 20, which is the number of infections when the simulations began. Any transmission rate lower than 0.05 but greater than zero produces a curve that lies between the curve in Figure Three (in reference [25]) and the straight line with intercept of 20 and slope of zero. As the transmission rate falls below 0.05, the curve must shift downward (the intercept falls from 120 to a minimum of 20), but also flatten out. The smaller slope means that the contrast in HIV infections between concurrency and serial monogamy is smaller. Moreover, for any decrease in the transmission rate, the slope must fall faster, the higher the level of concurrency. Epstein's mathematics of concurrent partnerships, transmission rates and HIV is incorrect.

This demonstration would be unnecessary if Morris and Kretzschmar would simply publish the results of simulations of their model using lower, realistic transmission rates. The first published criticism of their choice of transmission rate appeared in 2007 [28] and there has been ample time to produce new simulations using parameters that do not make their results so misleading for HIV-prevention policy.

## Appendix C

With a transmission rate of 0.05 and daily sex, the probability that HIV will spread from an infected person to an uninfected partner in six weeks is 88.4% [= 100 × (1 - (1 - 0.05)^42^)]. Most estimates of transmission rates during acute infection are between 0.01 or 0.02 [63,64] and those rates with daily sex produce a probability of transmission of 34% to 57% in six weeks. In contrast, with sex every 10 days for six weeks and a transmission rate of 0.01 to 0.02, the probability of transmission falls to between 4% and 8%. With a transmission rate of 0.001 after acute infection, sex every 10 days for a year produces less than a 4% chance of transmission.

## Appendix D

Kapiga and Lugalla [67] report concurrency for unmarried, previously married, and married/cohabiting adults (Table Two, page 459, in reference [67]). Average concurrency for all adults is calculated using weights from Table Two, page 460, and Table Three, page 462, in reference [67].

## Competing interests

The authors declare that they have no competing interests.

## Authors' information

Larry Sawers is Professor of Economics at American University. Eileen Stillwaggon is Professor of Economics and Harold G. Evans-Eisenhower Professor at Gettysburg College.

## Authors' contributions

LS and ES read the articles, books and papers reviewed here and collaborated in writing the article. Both authors have read and approved the final manuscript.

## Supplementary Material

Additional file 1 Table S1- Quantitative studies cited by Halperin, Epstein and Mah: Reasons why they do not support the concurrency hypothesis. Click here for file
