# Supplementary material for: Concurrent sexual partnerships do not explain the HIV epidemics in Africa: a systematic review of the evidence
Source: J Int AIDS Soc. 2010 Sep 13;13:34. doi: 10.1186/1758-2652-13-34 (PMC3161340; doi:10.1186/1758-2652-13-34)
Supplement: Additional file 1 — Table S1- Quantitative studies cited by Halperin, Epstein and Mah: Reasons why they do not support the concurrency hypothesis. [file 1758-2652-13-34-S1.PDF]

Table S1. **Quantitative studies cited by Halperin, Epstein and Mah: Reasons why they do not support the concurrency hypothesis.**

Studies (33)	Study no.	Study does not support concurrency hypothesis	Number of reasons why study does not support concurrency hypothesis	Halperin, Epstein, Mah incorrectly report results of study	Study reports behavior that is neither broadly nor narrowly defined concurrency	Study not relevant for other reasons	Halperin, Epstein, Mah do not report concurrency narrowly defined although reported in the study	Reported data cannot be compared to other studies (restricted age, race, sexual experience, marital groups, not all adults)	Data from study cannot be used to make statistical inferences about national populations			Questionnaire design not recommended by UNAIDS and exaggerates concurrency	Study reports concurrency low in Africa or high elsewhere	Citation insufficient to locate study
									Study of sub-national area	Study of high risk area or group	Study uses non-random sample or small sample			
Adimora *et al*, 2002 [71]	1	*	2	*									*	
Adimora *et al*, 2004 [70]	2	*	5	*		*		*	*		*			
Adimora *et al*, 2007 [72]	3	*	2	*									*	
Caldwell *et al*, 1999 [86]	20	*	4	*				*	*		*			
Campbell *et al* 2009 [74]	5	*	2	*		*								
Caraël, 1995 [46]	32	*	1						3 of 11 surveys			*		
Caraël *et al*, 2001 [94]	33	*	2						*			*		
Carter *et al*, 2007 [88]	25	*	5	*			*	*	*			*	*	
Colvin *et al*, 1998 [82]	12	*	4		*				*		*	*		
Drumright *et al*, 2004 [79]	9	*	5			*		*	*		**			
Ferguson *et al*, 2004 [91]	20	*	6					*	*	*	**	*	*	
Gourvenec *et al*, 2007 [53]	19	*	7	***			*	*				*	*	
Gras *et al*, 1997 [73]	4	*	7	*		*		*	*	*	*	*		
Gregson *et al*, 2002 [78]	7	*	2			*			*		.			
Guwatudde *et al*, 2009 [77]	6	*	3			*			*		*			
Harrison *et al*, 2008 [47]	23	*	3					*	*			*		
James *et al*, 2006 [NA]	13	*	3	NA	*			NA	*	NA	NA	NA		*
Kapiga *et al*, 2002 [67]	28	*	2									*	*	
Koumans *et al*, 2001 [80]	10	*	3			*			*		*			
Leridon *et al*, 1998 [93]	31	*	1										*	
Lesotho Health Survey [51]	14	*	5	***	*			*						
Mah, 2010 [69]	24	*	3		NA			*	*			*		
Mattson *et al*, 2007 [44]	22	*	5	*				*	*	*	*			
Meyerson *et al*, 2003 [89]	26	*	4	*				*	*			*		
Morris, NIH meeting [NA]	17	*	1	NA							NA			*
Morris, ASA meetings [NA}	18	*	1	NA							NA			*
Mwaluko *et al*, 2003 [90]	27	*	2		*				*					
Parker, Connolly, 2007 [85]	15	*	2		*				*					
Parker *et al*, 2007 [84]	16	*	2		*				*					
Potterat *et al*, 1999 [81]	11	*	2			*			*					
Shisana, *et al*, 2005 [49]	8	*	3	*	NA			*						
Voeten *et al*, 2004 [87]	21	*	5	**				*	*	*				
Williams *et al*, 2003 [92]	30	*	5		*			*	*	*	*			
TOTAL		33	109	18	7	8	2	15	25	5	10	11	7	3
